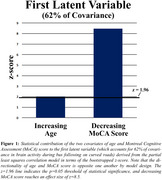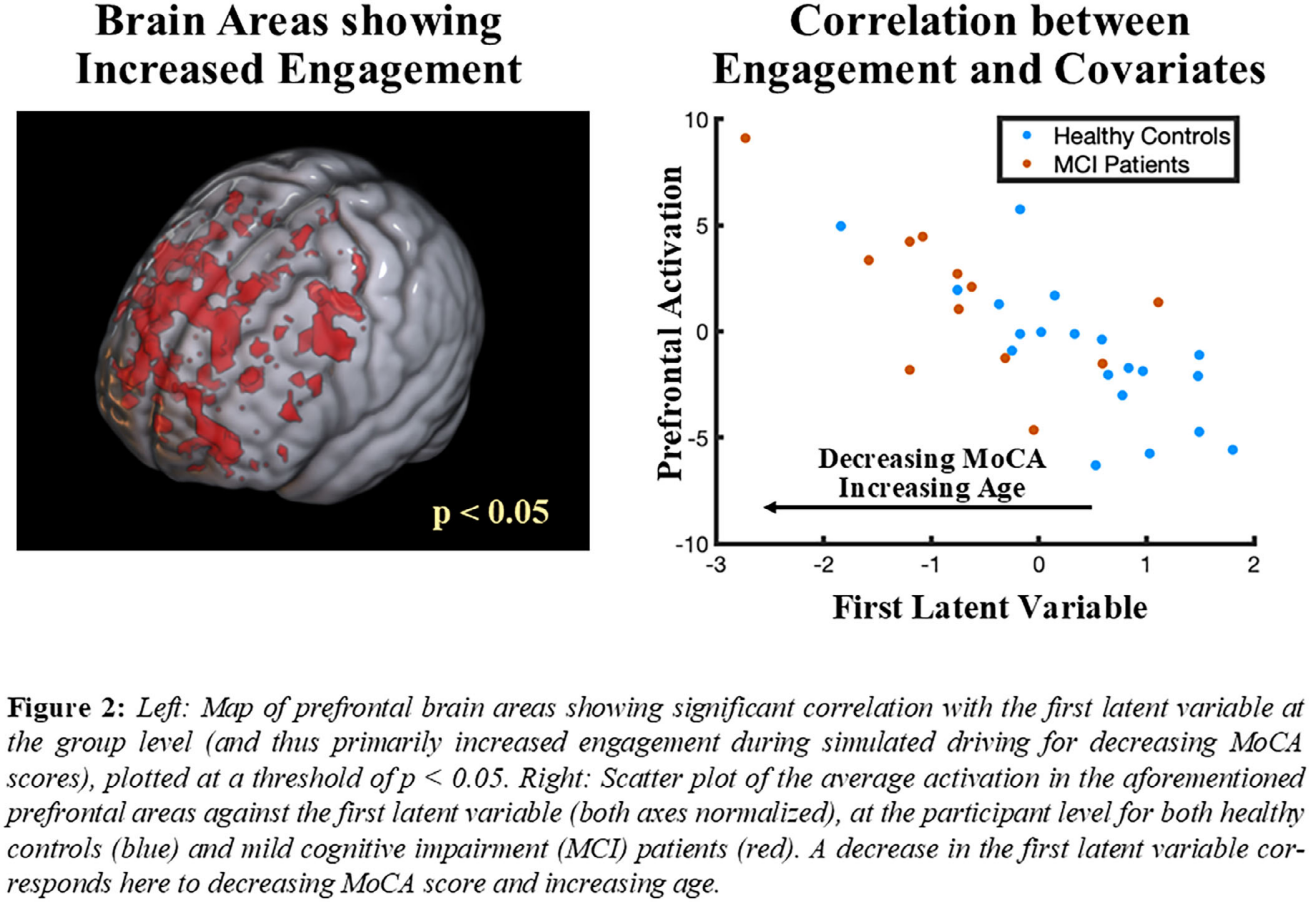# The Influence of Cognitive Status on Brain Activity during Simulated Driving

**DOI:** 10.1002/alz70856_100403

**Published:** 2025-12-25

**Authors:** Felix Menze, Nathan W. Churchill, Corinne E. Fischer, Simon J. Graham, Tom A. Schweizer

**Affiliations:** ^1^ University of Toronto, Toronto, ON, Canada; ^2^ Sunnybrook Health Sciences Centre, Toronto, ON, Canada; ^3^ Keenan Research Centre for Biomedical Science, Li Ka Shing Knowledge Institute, St. Michael's Hospital, Toronto, ON, Canada; ^4^ Sunnybrook Research Institute, Toronto, ON, Canada; ^5^ University of Toronto, Torono, ON, Canada; ^6^ Division of Neurosurgery, St. Michael's Hospital, Toronto, ON, Canada

## Abstract

**Background:**

Driving with mild cognitive impairment (MCI) or early‐stage Alzheimer's disease is common in many countries, despite the substantially increased risk of fatal accidents. In of Ontario, Canada, the government mandates that elderly drivers over 80 take cognitive screening tests (CSTs) every two years. However, there is little evidence to date that CST scores predict changes in the neural mechanisms underlying driving.

The present work addresses this void by studying the correlation of brain activity during a simulated bus‐following task with results of the Montreal Cognitive Assessment (MoCA) in a cohort of older drivers with and without MCI. It is hypothesized that drivers with low MoCA scores show increased activity of prefrontal areas (typically engaged during complex driving) when successfully performing bus following (staying in lane while following at safe distance on curved roads). Confirming these assertions would constitute early supporting evidence that simulated driving performance is modulated by cognitive status.

**Methods:**

Thirty‐two licensed drivers (50‐76yrs, 28% female) were recruited from patients diagnosed with MCI (*N* = 12; MoCA: 22‐27) and age‐matched controls (*N* = 20; MoCA: 24‐29). Participants performed a simulated bus‐following task (∼220s) during fMRI at 3.0 Tesla, which required navigation of multiple roads while keeping a constant distance to a leading bus driving at variables speed. Brain activity associated with curved segments was estimated using participant‐level general linear models, and subsequently correlated with MoCA score and age at group level using partial least squares analysis.

**Results:**

Sixty‐two percent of the covariance in brain activation during bus following was explained by a latent variable combining increasing age (bootstrapped z‐score=2.0) and decreasing MoCA score (z‐score=8.5; Figure 1). This latent variable correlated with increased brain activity in widespread prefrontal areas (Figure 2), including medial superior prefrontal cortex and dorsolateral superior and medial prefrontal cortex.

**Conclusions:**

The present study showed, for the first time, correlations between brain activity evoked during simulated safe driving and MoCA scores. Participants of lower MoCA score showed increased engagement of widespread prefrontal area, commonly associated with sustained attention and planning. Future work will design an adaptive test battery specifically screening for declines in fitness to drive.